# Supplementary figures and images for: Adenylate kinase 1 overexpression increases locomotor activity in medaka fish
Source: PLoS One. 2022 Jan 4;17(1):e0257967. doi: 10.1371/journal.pone.0257967 (PMC8726475; doi:10.1371/journal.pone.0257967)

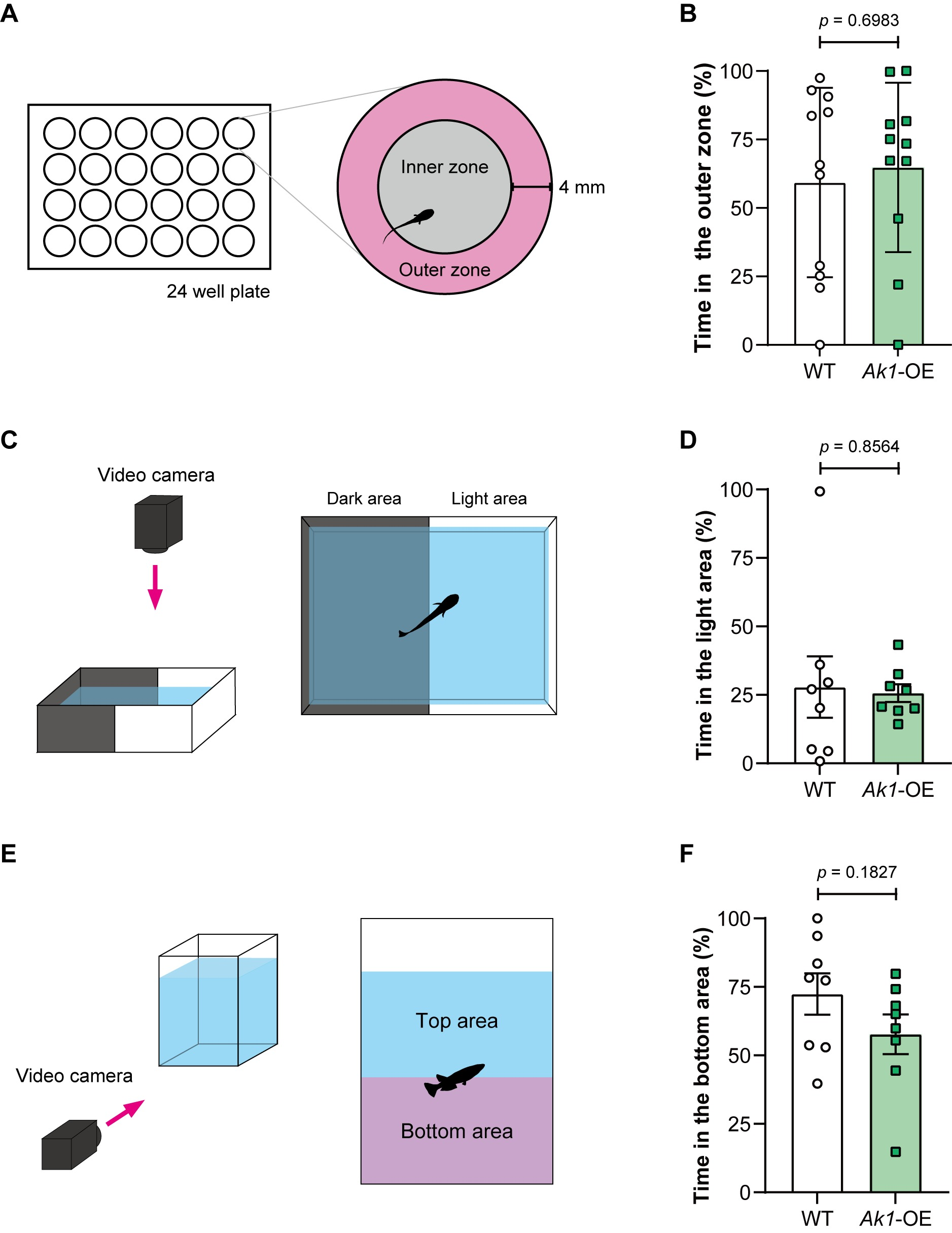

Supplement: S1 Fig — (A) Schematic drawing of thigmotaxis measurement test. (B) Results of thigmotaxis measurement test (n = 11 each). (C) Schematic of light-dark tank test. (D) Results of light-dark tank test (n = 8 each). (E) Schematic representation of the novel tank test. (F) Results of the novel tank test (n = 8 each). All data are presented as the mean ± SEM. The p-value was calculated using a two-tailed Welch’s t-test. Each dot represents an individual value. (TIF) [file pone.0257967.s001.tif]
