# Supplementary material for: Adenylate kinase 1 overexpression increases locomotor activity in medaka fish
Source: PLoS One. 2022 Jan 4;17(1):e0257967. doi: 10.1371/journal.pone.0257967 (PMC8726475; doi:10.1371/journal.pone.0257967)
Supplement: S1 Raw images — (PDF) [file pone.0257967.s002.pdf]

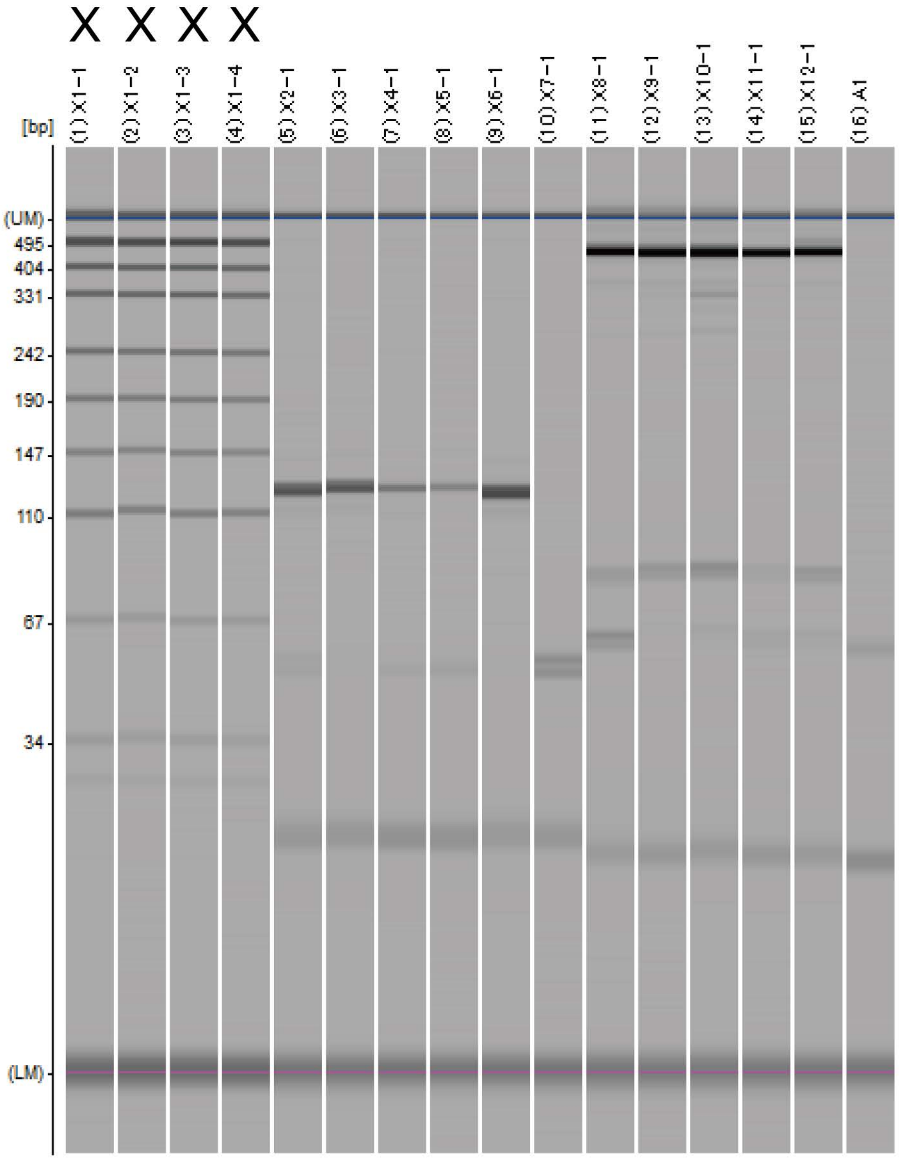

Fig 1B Raw image

Equipment: MultiNA microchip electrophoresis system (Shimadzu Corporation, Kyoto, Japan)

(1)-(4) pUC19 DNA/MspI (HpaII) Marker (Thermo Fisher Scientific, Waltham, MA, USA)

(5) *Ak1* primers, Brain cDNA

(6) *Ak1* primers, Eye cDNA

(7) *Ak1* primers, Ovary cDNA

(8) *Ak1* primers, Liver cDNA

(9) *Ak1* primers, Skin cDNA

(10) *Ak1* primers, without cDNA

(11) *Actb* primers, Brain cDNA

(12) *Actb* primers, Eye cDNA

(13) *Actb* primers, Ovary cDNA

(14) *Actb* primers, Liver cDNA

(15) *Actb* primers, Skin cDNA

(16) *Actb* primers, without cDNA

Ak1: 114 bp, *Actb*: 432 bp

Lanes not included in Fig 1B are marked with an “X” above the lane label.

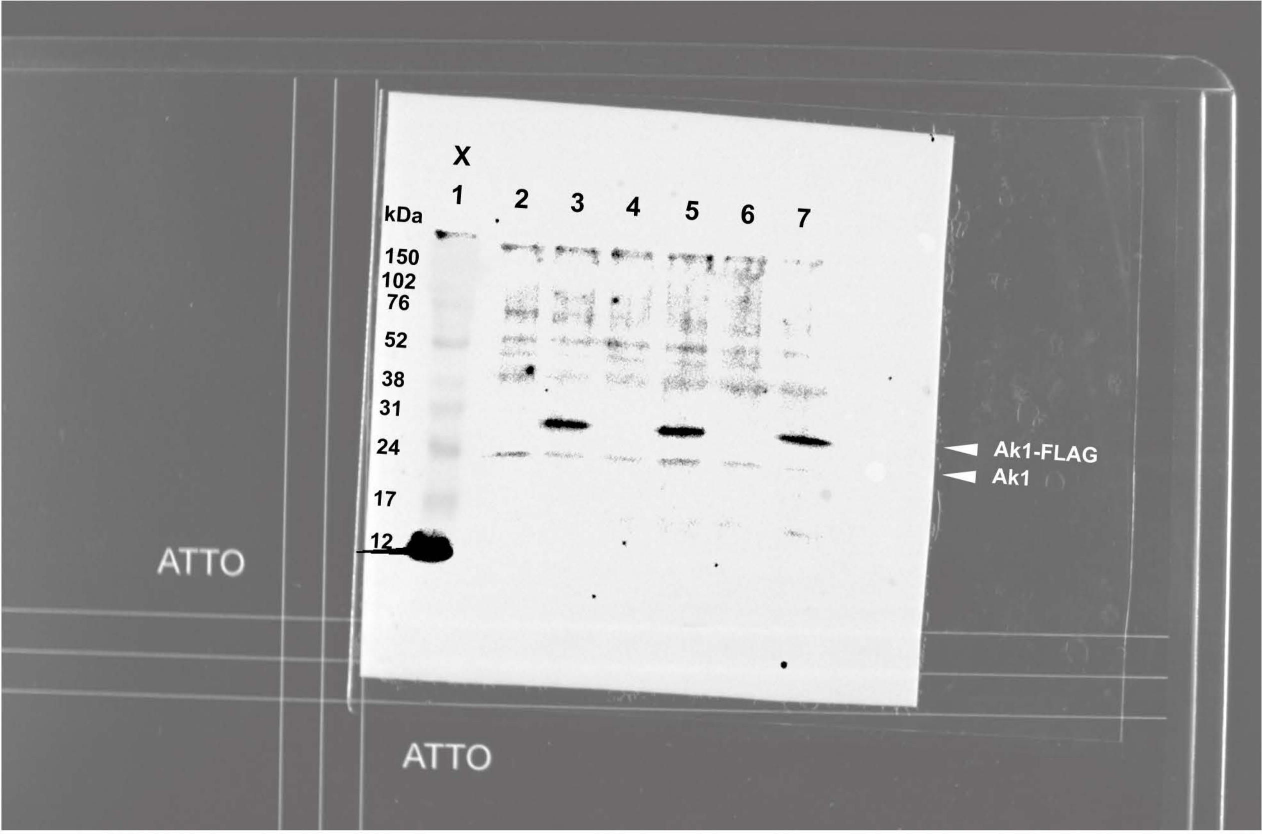

Fig. 1G (left) Raw image

Equipment: LuminoGraph II EM (ATTO, Tokyo, Japan)

Lane (1): ECL Rainbow Marker-Full Range (GE Healthcare, Chicago, IL, USA)

Lanes (2), (4), (6) WT medaka larvae, whole bodies

Lanes (3), (5), (7) *Ak1*-OE medaka larvae, whole bodies

Protein at 25 μg per lane.

Primary antibody:

AK1 polyclonal Antibody (14978-1-AP; Proteintech, Rosemont, IL, USA) at 1/500 dilution

Secondary antibody:

Anti-Rabbit IgG, HRP-Linked Whole Ab Donkey (NA934-100UL; GE Healthcare, Chicago, IL, USA) at 1/1000 dillution

Lanes not included in Fig 1G are marked with an “X” above the lane label.

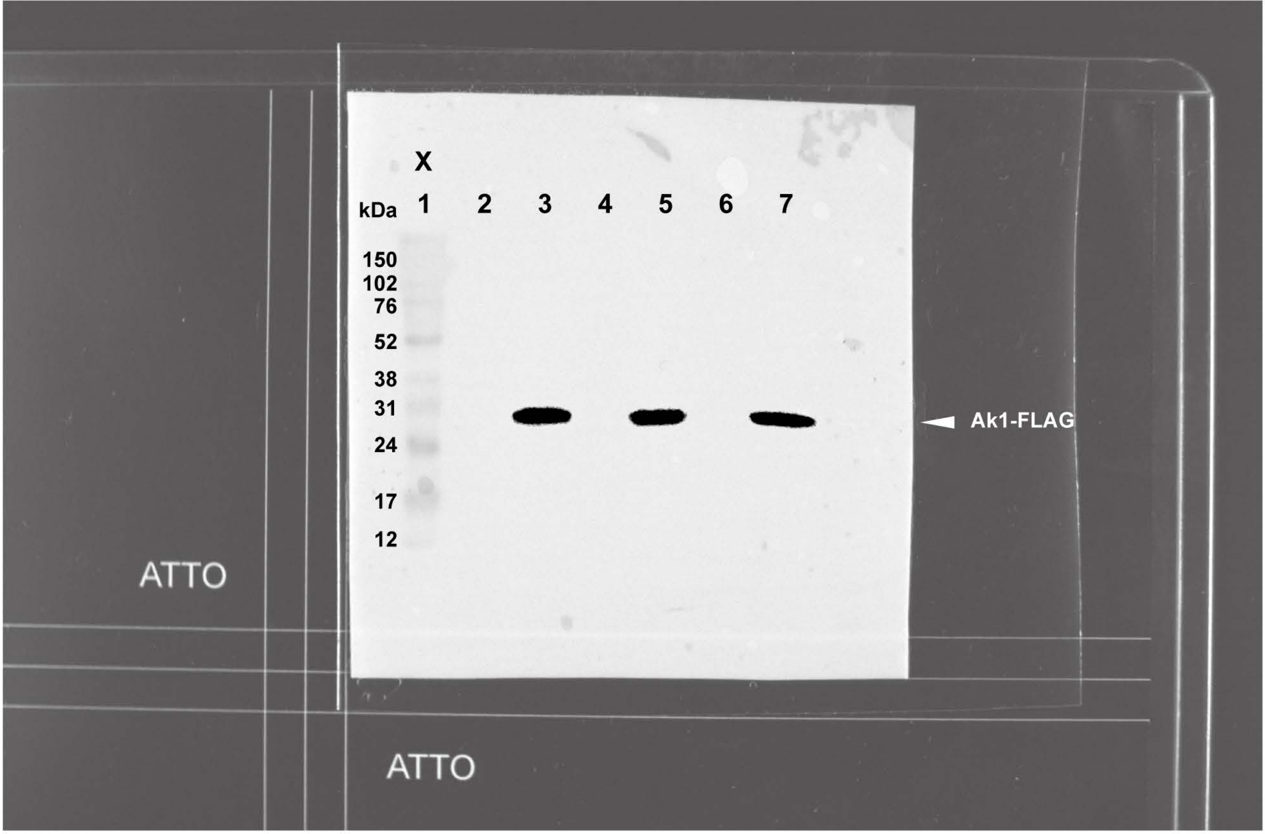

Fig 1G (right) Raw image

Equipment: LuminoGraph II EM (ATTO, Tokyo, Japan)

Lane (1): ECL Rainbow Marker-Full Range (GE Healthcare, Chicago, IL, USA)

Lanes (2), (4), (6) WT medaka larvae, whole bodies

Lanes (3), (5), (7) *Ak1*-OE medaka larvae, whole bodies

Protein at 25 μg per lane.

Primary: Anti-DDDDK-tag mAb (M185-3S; MBL, Nagoya, Japan) at 1/1000 dilution

Secondary: Anti-Mouse IgG, HRP-Linked Whole Ab Sheep (NA931-100UL; GE Healthcare) at 1/1000 dillution

Lanes not included in Fig 1G are marked with an “X” above the lane label.
