# Supplementary material for: Adenylate kinase 1 overexpression increases locomotor activity in medaka fish
Source: PLoS One. 2022 Jan 4;17(1):e0257967. doi: 10.1371/journal.pone.0257967 (PMC8726475; doi:10.1371/journal.pone.0257967)
Supplement: Attachment — Submitted filename: Response_to_Reviewers.docx [file pone.0257967.s003.docx]

**Point-by-point responses to the reviewers’ comments to Maruyama et al., “Adenylate kinase 1 overexpression increases locomotor activity in medaka fish”**

We are grateful to the reviewers for their positive and valuable comments, which have helped us improve our manuscript. The reviewers’ comments are shown in blue font below, and our responses are shown in black font.

Reviewer #1: This manuscript by Maruyama and colleagues addresses an interesting issue regarding the links between cellular energy metabolism and behavioural activity. Using a medaka transgenic model, the authors study the consequences of overexpression of one key isoform of the enzyme Adenylate kinase (AK1) on locomotor activity of larval medaka following Day 8 post fertilization. The overall basal level of locomotor activity measured in the transgenics appears to be significantly higher than in the wild type controls, but only when measured at 25°C. Instead, during the locomotor activity assay performed at 15°C, activity of the wild type and transgenics were indistinguishable.

This is an interesting observation that deserves to be published, however I would propose the following modifications:

1) It is well known that in teleosts, many gene families are larger than in mammals due to the effect of extra gene copies resulting from ancestral genome duplication events. This leads to the obvious question: are there only 9 isoforms of AK in medaka? If there are more, it would be helpful to include a figure with a phylogenetic tree comparing the mammalian and medaka situation.

**Response**: Only Ak7 was duplicated in medaka. As suggested, we have added the phylogenetic tree in Fig. 1A. Accordingly, we have changed the figure order.

We have also added the following text in the Methods section:

**Phylogenetic analysis**

The phylogenetic tree was constructed using the maximum likelihood method with MEGA 11 (https://www.megasoftware.net/). The GenBank accession numbers of the nucleotide sequences used for the phylogenetic analysis are as follows:

Human (*Homo sapiens*) *AK1*, NM_000476.3; Mouse (*Mus musculus*) *Ak1*, NM_001198790.1; Medaka (*Oryzias latipes*) *Ak1*, XM_004074742.4; Human *AK2*, NM_001199199.3; Mouse *Ak2*, NM_001033966.4; Medaka *Ak2*, XM_011481017.3; Human *AK3*, NM_001199852.2; Mouse *Ak3*, NM_001365071.1; Medaka *Ak3*, XM_004074841.4; Human *AK4*, NM_001005353.3; Mouse *Ak4*, NM_001177602.1; Medaka *Ak4*, XM_004068018.4; Human *AK5*, NM_012093.4, Mouse *Ak5*, NM_001081277.2; Medaka *Ak5*, XM_004078698.4; Human *AK6*, NM_001015891.2; Mouse *Ak6*, NM_027592.3; Medaka *Ak6*, XM_004072821.3; Human *AK7*, NM_001350888.2; Mouse *Ak7*, NM_030187.1; Medaka *Ak7*, XM_011490546.3; Medaka LOC101157414, XM_011492034.2; Human *AK8*, NM_001317958.2; Mouse *Ak8*, NM_001033874.2; Medaka *Ak8*, XM_023958761.1; Human *AK9*, NM_001145128.3; Mouse *Ak9*, NM_001370813.1; Medaka *Ak9*, XM_020714548.2

2) While it is clear that the AK1 transgene is expressed from a strong, widely expressed promoter, there is no evidence provided comparing AK1 protein levels or AK1 enzymatic activity in the transgenics and wild types. This would be particularly important information to fully interpret the comparison of the behaviour at the two different temperatures. If technically feasible, such extra information would be invaluable for interpreting the results.

**Response**: We determined Ak1 protein levels in both *Ak1*-OE and WT by western blot analysis using the Ak1 antibody. The results are as follows:

In both *Ak1*-OE and WT samples, we observed bands close to the expected band size of medaka Ak1 (21.4 kDa). In addition, we found strong bands at high molecular weights only in *Ak1*-OE samples. In our overexpression construct, we added a 3× FLAG tag downstream of the Ak1 sequence. Therefore, we expected that the strong bands observed were FLAG-tagged Ak1. To confirm this, we conducted western blot analysis using a FLAG-tag antibody. The results are as follows:

As expected, we observed bands at the same position as those detected with the Ak1 antibody. From these results, we confirmed Ak1 overexpression at both the gene expression and protein levels. We have added these results in the revised manuscript.

We have also added the following text in the Methods section:

**Western blot analysis**

Frozen 9 dpf larvae were homogenized and sonicated in 4× Laemmli sample buffer (Bio-Rad, Hercules, CA, USA). Samples were quantified using Pierce 660 nm Protein Assay Reagent (Thermo Fisher Scientific, Waltham, MA, USA). Twenty-five micrograms of protein per sample were separated by SDS-PAGE and transferred onto a PVDF membrane. The PVDF membrane was blocked with 5% skim milk in Tris-buffered saline with Tween 20 (TBST) for 1 h at room temperature. Ak1 polyclonal primary antibody (1:500) (14978-1-AP; Proteintech, Rosemont, IL, USA) was dissolved in 5% skim milk in TBST and incubated for 1 h at room temperature. After washing the membrane with TBST, the membranes were incubated with secondary rabbit antibody (1:1000) (NA934-100UL; GE Healthcare, Chicago, IL, USA) dissolved in 5 % skim milk in TBST for 1 h at room temperature. The membrane was washed with TBST and incubated with ECL prime (GE Healthcare) for 5 min at room temperature and imaged on a LuminoGraph II EM (ATTO, Tokyo, Japan). After eliminating signals using hydrogen peroxide, the membrane was treated with FLAG (DDDDK) tag primary monoclonal antibody (1:1000) (M185-3S; MBL, Nagoya, Japan) followed by secondary mouse antibody (1:1000) (NA931-100UL; GE Healthcare).

Reviewer #2: This manuscript by Maruyama et al. describes the effect of adenylate kinase 1 overexpression on locomotor activity in medaka. The authors found that Ak1-OE increases locomotor activity at a water temperature of 25C in the light period. As such a change did not occur at 15C, the authors suggested the possibility of modulating locomotor activity by Ak1 depending water temperature. This MS was short and well written about well-designed experiments. I have only one question, and changes in locomotor activity may reflect some change in taxis. Did the authors observe any changes in thigmotaxis, scototaxis, geotaxis, etc.

**Response**: To test whether the increase in locomotor activity of *Ak1*-OE was due to changes in some taxis, we conducted several additional behavioral tests. When we examined changes in thigmotaxis between *Ak1*-OE and WT as described by Schnorr et al. [18], no difference was observed. We also conducted a light-dark tank test and a novel tank test for scototaxis and geotaxis, respectively. However, we found no difference between *Ak1*-OE and WT in these tests, suggesting that Ak1 indeed affects locomotor activity but not taxis. We have added these results to S1 Fig as follows:

S1 Fig. Comparison of thigmotaxis, scototaxis, and geotaxis between *Ak1*-OE and WT.

(A) Schematic drawing of thigmotaxis measurement test. (B) Results of thigmotaxis measurement test (n = 11 each). (C) Schematic of light-dark tank test. (D) Results of light-dark tank test (n = 8 each). (E) Schematic representation of the novel tank test. (F) Results of the novel tank test (n = 8 each). All data are presented as the mean ± SEM. The *p*-value was calculated using a two-tailed Welch’s *t*-test. Each dot represents an individual value.

We have also added the following text in the Methods section:

To examine thigmotaxis, we referred to Schnorr et al. [18]. In brief, larvae were transferred to a 24-well round plate (Nunclon Delta Surface; Thermo Fisher Scientific, Waltham, MA, USA) at 8 dpf. The plate was then installed in a DanioVision Observation Chamber at 9 dpf. After 6 min of acclimation phase (light on), the lights were kept off for 4 min and activities were recorded. The experiments were conducted from 10:00 to 12:00 at 9 dpf.

To examine scototaxis and geotaxis, we conducted a light-dark tank test and a novel tank test, respectively [19]. In these assays, adult fish (> 6 months) were used. For the novel tank test, each fish was introduced into a glass tank (100 mm width × 65 mm depth × 142 mm height) filled with water to a height of 100 mm. Recordings were started immediately after transferring the fish and lasted for 7 min. We excluded the first 1 min from the analysis. For the light-dark tank test, a plastic container (150 mm width × 100 mm depth × 55 mm height) was used. The container was divided into two equal-sized sections, colored either white or black. Fish were placed in the light area and allowed to move freely between the dark and light areas for 16 min. We excluded the first 1 min from the analysis.

**Response to Academic Editor:**

1. Please ensure that your manuscript meets PLOS ONE's style requirements, including those for file naming. The PLOS ONE style templates can be found at

https://journals.plos.org/plosone/s/file?id=wjVg/PLOSOne_formatting_sample_main_body.pdf and

https://journals.plos.org/plosone/s/file?id=ba62/PLOSOne_formatting_sample_title_authors_affiliations.pdf

**Response**: We have revised the manuscript according to the guidelines of PLOS ONE as suggested.

2. To comply with PLOS ONE submissions requirements, in your Methods section, please provide additional information on the animal research and ensure you have included details on (1) methods of sacrifice, (2) methods of anesthesia and/or analgesia, and (3) efforts to alleviate suffering.

**Response**: We added the following text on “Ethics statement for animal experiments” in the Methods section:

Animals were euthanized with ethyl 3-aminobenzoate methanesulfonate (MS-222) before sampling. All efforts were made to minimize animal suffering.

3. Thank you for stating in your Funding Statement:

[This work was supported in part by a JSPS KAKENHI “Grant-in-Aid for Scientific Research (S)” (19H05643) for T.Y. and the Sasakawa Scientific Research Grant from The Japan Science Society for M.M.]

Please provide an amended statement that declares *all* the funding or sources of support (whether external or internal to your organization) received during this study, as detailed online in our guide for authors at http://journals.plos.org/plosone/s/submit-now. Please also include the statement “There was no additional external funding received for this study.” in your updated Funding Statement.

Please include your amended Funding Statement within your cover letter. We will change the online submission form on your behalf.

**Response**: We have changed the original statement to:

This work was supported by a JSPS KAKENHI “Grant-in-Aid for Scientific Research (S)” (19H05643) for T.Y. and the Sasakawa Scientific Research Grant from The Japan Science Society for M.M. There was no additional external funding received for this study. The funders had no role in the study design, data collection and analysis, decision to publish, or preparation of the manuscript.

4. In your Data Availability statement, you have not specified where the minimal data set underlying the results described in your manuscript can be found. PLOS defines a study's minimal data set as the underlying data used to reach the conclusions drawn in the manuscript and any additional data required to replicate the reported study findings in their entirety. All PLOS journals require that the minimal data set be made fully available. For more information about our data policy, please see http://journals.plos.org/plosone/s/data-availability.

Upon re-submitting your revised manuscript, please upload your study’s minimal underlying data set as either Supporting Information files or to a stable, public repository and include the relevant URLs, DOIs, or accession numbers within your revised cover letter. For a list of acceptable repositories, please see http://journals.plos.org/plosone/s/data-availability#loc-recommended-repositories. Any potentially identifying patient information must be fully anonymized.

Important: If there are ethical or legal restrictions to sharing your data publicly, please explain these restrictions in detail. Please see our guidelines for more information on what we consider unacceptable restrictions to publicly sharing data: http://journals.plos.org/plosone/s/data-availability#loc-unacceptable-data-access-restrictions. Note that it is not acceptable for the authors to be the sole named individuals responsible for ensuring data access.

We will update your Data Availability statement to reflect the information you provide in your cover letter.

**Response**: We have amended as follows:

All relevant data are within the manuscript and its Supporting Information files.

5. PLOS ONE now requires that authors provide the original uncropped and unadjusted images underlying all blot or gel results reported in a submission’s figures or Supporting Information files. This policy and the journal’s other requirements for blot/gel reporting and figure preparation are described in detail at https://journals.plos.org/plosone/s/figures#loc-blot-and-gel-reporting-requirements and https://journals.plos.org/plosone/s/figures#loc-preparing-figures-from-image-files. When you submit your revised manuscript, please ensure that your figures adhere fully to these guidelines and provide the original underlying images for all blot or gel data reported in your submission. See the following link for instructions on providing the original image data: https://journals.plos.org/plosone/s/figures#loc-original-images-for-blots-and-gels.

In your cover letter, please note whether your blot/gel image data are in Supporting Information or posted at a public data repository, provide the repository URL if relevant, and provide specific details as to which raw blot/gel images, if any, are not available. Email us at plosone@plos.org if you have any questions.

**Response**: We have uploaded the blot/gel image file (S1_raw_images) as a Supporting Information file.
